# Disruption of Cerebellar Prediction in Verbal Working Memory

**DOI:** 10.3389/fnhum.2019.00061

**Published:** 2019-02-21

**Authors:** Yi-Shin Sheu, Yu Liang, John E. Desmond

**Affiliations:** Department of Neurology, Division of Cognitive Neuroscience, Johns Hopkins University School of Medicine, Baltimore, MD, United States

**Keywords:** cerebellum, TMS, verbal working memory, forward models, prediction

## Abstract

Mounting evidence suggests that the right cerebellum contributes to verbal working memory, but the functional role of this contribution remains unclear. In an established theory of motor control, the cerebellum is thought to predict sensory consequences of movements through an internal “forward model.” Here, we hypothesize a similar predictive process can generalize to cerebellar non-motor function, and that the right cerebellum plays a predictive role that is beneficial for rapidly engaging the phonological loop in verbal working memory. To test this hypothesis, double-pulse transcranial magnetic stimulation (TMS) was administered over either the right cerebellum or right occipital lobe (control site), on half the trials, to interrupt the rehearsal of a 6-letter sequence. We found that cerebellar stimulation resulted in greater errors in participants’ report of the letter in the current position. Additional analyses revealed that immediately after cerebellar TMS, participants were more likely to use out of date information to predict the next letter in the sequence. This pattern of errors is consistent with TMS causing a temporary disruption of state estimation and cerebellar forward model function, leading to prediction errors in the phonological loop.

## Introduction

It has become widely accepted in recent years that the human cerebellum contributes not only to motor function, but also to a wide range of non-motor cognitive functions (for reviews, see Stoodley, 2012; Buckner, 2013; Schmahmann, 2019), such as verbal working memory (Chein and Fiez, 2001; Chen and Desmond, 2005; Justus et al., 2005; Ravizza et al., 2006; Hayter et al., 2007; Durisko and Fiez, 2010; Marvel and Desmond, 2010; Peterburs et al., 2010, 2016; Stoodley et al., 2012), executive function (Grafman et al., 1992; Rao et al., 1997; Schmahmann and Sherman, 1998; Karatekin et al., 2000; Neau et al., 2000; Bellebaum and Daum, 2007; Balsters et al., 2013; Wu et al., 2013; Rentiya et al., 2017), and language (Petersen et al., 1989; Desmond et al., 1998; Fulbright et al., 1999; Leggio et al., 2000; Lurito et al., 2000; Seger et al., 2000; Moretti et al., 2002; Xiang et al., 2003; Grönholm et al., 2005; Frings et al., 2006; Ben-Yehudah and Fiez, 2008; Rauschecker et al., 2008; Mariën et al., 2009, 2014; Stoodley and Schmahmann, 2009; Highnam and Bleile, 2011; Argyropoulos and Muggleton, 2013; Keren-Happuch et al., 2014). However, the nature of cerebellar contributions to these cognitive functions remains unclear.

Working memory, the ability to temporarily store and manipulate information for complex cognitive activities (Baddeley, 1998), is perhaps one of the most studied cognitive function that engages cerebellum. Based on the theoretical framework of working memory by Baddeley and Hitch (1974), a central executive system with limited attentional capacity is served by two subsidiary storage systems: the phonological loop for verbal information and the visuospatial sketchpad for visual information. The phonological loop comprises a phonological store, which can hold memory traces for a few seconds before they fade, and an articulatory rehearsal process that can refresh the memory trace through active rehearsal, which is analogous to sub-vocal speech (Baddeley, 1992). Previous neuroimaging studies of verbal working memory suggest that regions in left inferior temporal/parietal regions are associated with the phonological store, and the left inferior frontal regions are associated with articulatory control process (Paulesu et al., 1993; Awh et al., 1996; Fiez et al., 1996). Based on the known neuroanatomy of cerebro-cerebellar pathways (Middleton and Strick, 1994, 1997, 2001) and the use of a phase-specific Sternberg task (Sternberg, 1966), Desmond et al. (1997) proposed a neuroanatomical model of two cerebro-cerebellar circuits participating in the phonological loop: one connecting the frontal cortex to the superior cerebellum, providing the articulatory rehearsal process for phonological encoding, and the other connecting the temporal/parietal cortex to the inferior cerebellum, providing temporary maintenance of phonological information. This model was supported by subsequent functional neuroimaging studies (Chen and Desmond, 2005; Kirschen et al., 2010), cerebellar patient studies (Silveri et al., 1998; Ravizza et al., 2006; Chiricozzi et al., 2008; Kirschen et al., 2008; Peterburs et al., 2010), cerebellar transcranial magnetic stimulation (TMS; Desmond et al., 2005) and transcranial direct current stimulation (tDCS; Boehringer et al., 2013) investigations.

In the literature of motor control, forward models have been postulated as the basic computation provided by the cerebellum in order to control the musculoskeletal system, especially for rapid movements when sensory feedback delay is unavoidable (Wolpert and Miall, 1996; Wolpert et al., 1998). Forward models are essentially internal “neural” models that mimic the motor apparatus, which provide predictions of the sensory consequences of movements before feedback is available. Given the homogeneous cytoarchitecture of the cerebellar cortex, some investigators have argued that there is a common computational operation performed throughout the structure, with difference in function derived from the local input-output connections with the cerebral cortex (Ramnani, 2006; Ito, 2008; Strick et al., 2009; Bellebaum et al., 2012; Ishikawa et al., 2016). If the computational principles are indeed similar across the cerebellum, then our understanding of cerebellar function in sensorimotor control might be relevant to cerebellar involvement in verbal working memory. Therefore, in the current study, we propose the right cerebellum plays a predictive role, similar to forward models in motor control, that is beneficial for rapidly engaging the phonological loop in verbal working memory.

Given our cerebro-cerebellar model of phonological loop described earlier, we hypothesize that the cerebellum contributes to verbal working memory by generating two distinct predictions: (1) predictions of the articulatory trajectory based on the encoded verbal items, which may involve planning of movements of our jaw, tongue, lips, and larynx; and (2) predictions of the content in the phonological store, which may involve streaming a sequence of phonemes for sub-vocal rehearsal process. In a typical verbal working memory task, the success of correct verbal recall depends both on setting up an articulatory trajectory of the encoded verbal items as well as active rehearsal. Thus, we hypothesize that increased error rates in verbal working memory performance would occur if either the frontal/superior cerebellum articulatory prediction, or the parietal/inferior cerebellum phonological prediction, or both, were disrupted.

Direct evidence of disruption of cerebellar prediction in motor control has been observed using TMS, a brain stimulation technique that can temporarily interrupt function of the targeted area with high temporal specificity. Miall et al. (2007) tested the cerebellar forward model in a hand movement trajectory task by applying TMS to the cerebellum while participants made a rapid reaching movement toward a remembered target. This resulted in trajectory errors that could be explained by movements that were planned based on the hand position 138 ms ago. They suggested that the observed directional deviation was a result of a temporary loss of cerebellar predictive function, which caused the planning of reaching movement to be based on the previous (out of date) state of the arm.

Inspired by the Miall et al. (2007) results, we designed an analogous experiment to test our hypothesis that right cerebellum plays a predictive role in verbal working memory. We used TMS to briefly interfere with right cerebellar function as the participants covertly rehearsed a sequence of encoded letters. In order to generate articulatory trajectories with a known state over time, we used guided rehearsal, where a series of # signs, each representing a letter of the encoded sequence, was presented on the screen one at a time to pace the subject’s rehearsal of the letter sequence. This rehearsal process was interrupted by TMS, at which time the subject was immediately asked to report if a probe letter was the correct next-letter in the sequence. On half the trials the probe was the correct next letter, and on the other half the probe was either one letter earlier (early probe) or later (late probe) in the sequence. We predicted that, like the Miall et al. (2007) investigation, TMS would make the state estimation of the articulatory trajectory out of date (i.e., the forward models would be predicting a letter that was earlier in the trajectory instead of the correct next letter). Consequently, we predicted that cerebellar TMS would cause the participant to more likely judge an early probe as being in the correct position (more errors in early probe condition), a correct probe as being too late in the sequence (more errors in correct probe condition), and a late probe as (definitely) too late in the sequence (no or fewer errors). We used a control site in right occipital lobe to assess the specificity of cerebellar TMS effects.

## Materials and Methods

### Participants

A total of 23 (seven males, 16 females) healthy young adults, age 19–30 (mean = 22.26 years, SD = 2.649 years), with educational attainment of at least 8 years, participated in the study. All participants were native English speakers with normal or corrected-to-normal vision, had no history of head trauma, seizure or a family history of epilepsy, stroke, neurological or psychiatric disorders, and were not taking anxiolytic, antidepressant, neuroleptic, or sedative medication at the time of the study. This study was carried out in accordance with the recommendations of Institutional Review Board of the Johns Hopkins School of Medicine with written informed consent from all subjects. All subjects gave written informed consent in accordance with the Declaration of Helsinki. The protocol was approved by the Institutional Review Board of the Johns Hopkins School of Medicine.

### Tasks

**Figure 1:**
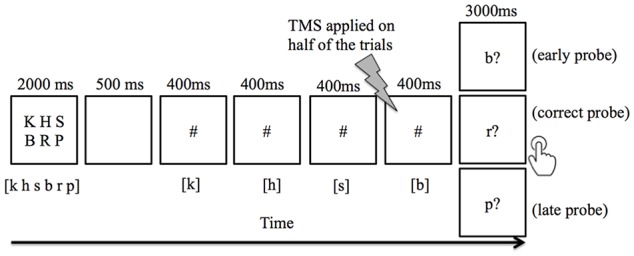
An example of trial events progression in the verbal working memory task. Subjects were instructed to keep in mind six visually-presented letters, and to covertly rehearse the letters in sync with the appearance of # symbols. Between each # presentation, a 150 ms blank screen was included to visually separate the adjacent # presentation. The letter(s) listed in [ ] indicates the correct content for rehearsal. When a probe letter appeared, the subject pressed button 1 to indicate that it matched the next letter in the sequence, or button 2 indicate that it did not match. The correct response for early probe and late probe conditions was the “non-match” button, whereas the “match” button was appropriate for the correct probe condition. Double-pulse transcranial magnetic stimulation (TMS) was applied 150 ms prior to the last # sign in half of the trials.

Participants were asked to covertly encode an array of six letters presented on a screen in uppercase, which was then removed from the screen after 2 s. Participants were instructed to read the letters in the order that they appeared (read from left to right, first row then second row). After a short delay (500 ms), 2–4 # signs then appeared on the screen one at a time (400 ms for each # sign with a 150 ms blank screen between # signs), each representing a placeholder of a letter in the encoding array. On half of the trials, participants received paired 20 Hz TMS pulses 150 ms prior to the last # sign, followed by a probe letter (3 s, presented in lowercase with a question mark. Participants were instructed to press button 1 for “yes” with their right index finger to indicate that the probe letter matches the next letter in the sequence, and to press 2 for “no” with their right middle finger if the probe letter does not match the next letter in the sequence (Figure 1). In addition, they were instructed to respond as fast as possible without sacrificing accuracy. The next trial began after a fixed interval of 1,800 ms. Participants were given a short practice at the beginning of the experiment so that they could familiarize themselves with the task. During the task practice, feedback was given to indicate whether the participant’s response was “Correct” or “Incorrect, ” and the accumulated percent accuracy was displayed. Feedback was not given during the actual TMS experiment.

A total of 80 trials were given for each session (cerebellum vs. occipital lobe stimulation, order counterbalanced). Eighty percent of these trials (*n* = 64) were longer trials with the probe letter appearing after the fourth # sign (i.e., probe at the fifth position = P5). The remaining 20% (*n* = 16) containing either 2 or 3 # signs (i.e., probe at the third or fourth position = P3 and P4) were discarded from analysis because a previous pilot study in our lab (unpublished) showed these shorter trials had a ceiling effect due to its short duration and thus lower working memory demands. However, the shorter trials were included to ensure that the timing of the probe letter was unpredictable. For both the longer trials (P5) and shorter trials (P3 and P4), there were an equal number of TMS and non-TMS trials as well as an equal number of match (i.e., correct probe) and non-match (i.e., early and late probe) trials.

### TMS Protocol

Biphasic TMS paired-pulses were triggered at 20 Hz (i.e., 50 ms interpulse-interval) by E-Prime 2.0 standard software (Psychological Software Tool, Pittsburg, PA, USA) using a Magstim Rapid^2^ stimulator (Magstim Co., Whitland Dyfed, UK) that was connected to a 110-mm diameter double cone coil. Motor threshold (MT) was determined for each participant as the minimal TMS intensity needed to evoke a visible muscle twitch in the right hand in 5 out of 10 trials upon stimulation of the left motor cortex. The coil was placed on the scalp with the handle held backward and with the coil current flowing in an upward direction at the juncture of the two loops of the coil. The optimal stimulation location and the orientation of the coil were marked on a fitting lycra swimming cap placed over participant’s head to ensure consistent coil positioning.

For cerebellum stimulation, the double cone coil was centered at 1 cm below and 3 cm to the right of the inion. This coil geometry and position were found to be ideal for stimulating lateral cerebellar gray matter with low probability of passing through occipital cortex (Hardwick et al., 2014). However, in this position, we found that individual variations in skull shape created a gap between the scalp surface and the double cone coil to a varying degree in our participants. Previous studies have found that the scalp-coil distance directly influences the magnitude of stimulator output needed to reach MT (Kozel et al., 2000; McConnell et al., 2001; Stokes et al., 2007). Specifically, using a 70 mm figure-eight coil, Stokes et al. (2007) found for every 1 mm distance, an additional ~2.8% of stimulator output was required to reach the same level of MT. However, to our knowledge, no studies have systematically manipulated the scalp-coil distance using a double-cone coil, which was designed for stimulating the deeper cortical areas typically at the depth of 3–4 cm from the scalp, as comparison to figure-eight coil at the depth of 2–2.5 cm (Lu and Ueno, 2017). Therefore, before TMS stimulation of the cerebellum, we measured the MT for each participant at varying scalp-coil distances by placing custom-made moldable plastic separators^1^, measuring 3 mm, 7 mm, and 10 mm in thickness between the scalp surface and the coil. This resulted in four scalp-coil measurements: 0 mm (base level), 3 mm, 7 mm, and 10 mm. We then entered our measurements (X = separator thickness in mm, Y = stimulator output needed to reach MT) into a linear regression equation to derive the slope and the constant. In order to measure the actual distance between the participant’s scalp and the double cone coil for cerebellar TMS, we measured the scalp-coil distance using seven cylindrical wooden sticks in different diameters (4.75 mm, 6.43 mm, 8.2 mm, 9.75 mm, 11.25 mm, 12.5 mm, 15.04 mm), after we positioned the participant in the TMS chair and placed the coil as close as possible to the scalp. The cylindrical stick that was the best fit between the scalp and coil was used to calculate the adjusted stimulator output using each individual’s linear regression equation. The adjusted output number for scalp-coil distance was then multiplied by 110% to ensure excitability of the right cerebellum.

For the right occipital (control) region, the coil was centered at 7 cm above and 3 cm to the right of the inion. In this position, we did not experience any scalp-coil distance issues in all of our participants. Therefore, the stimulator output was directly set to 110% of MT. At this intensity, no participants reported phosphenes during the experiment. Finally, we note that the distance between the scalp and the targeted cortex is greater for cerebellum than for the occipital lobe, and consequently, the occipital lobe overall likely received more stimulation than the cerebellum.

### Data Analysis

Error rate and reaction time (RT) were analyzed using SPSS version 24 (IBM Corp, Armonk, NY, USA). Differences in the percentage of error rate between TMS and non-TMS trials were calculated for each participant, separately for each cerebellar and occipital stimulation session. The same subtraction was performed for mean RT. Repeated-measures analysis of variances (ANOVAs) were then conducted on these differences to test for an interaction between stimulation sites (cerebellum vs. occipital lobe) and probe position (early, correct, late). Based on our hypothesis described in the “Introduction” section that cerebellar TMS would make the state estimation of the predicted sequence out of date, we predicted significantly higher error rates for early and correct probe, but not for the late probe. To test this *a priori* hypothesis, we conducted a planned comparison based on the predicted interaction between stimulation site (cerebellum = +1, control site = −1) and probe position (early probe = +1, correct probe = +1, late probe = −2), followed by three planned comparisons using paired *t*-tests to determine whether mean difference in error rate was significantly different between these two stimulation sites for each probe condition.

## Results

### TMS Coil-Scalp Distance and MT

The mean MT (gap = 0 mm) was 36.39% (SD = 5.813%). The mean slope for the linear regression was 0.709%/mm (SD = 0.23%/mm), which means an additional 0.709% of absolute simulator output was required for each 1 mm distance between the scalp and coil to reach the same level of MT excitability. For cerebellar stimulation, the average distance between coil and scalp was 9.464 mm (SD = 2.726 mm). The average absolute stimulator output applied at the stimulation site was 46.91% (SD = 7.096%) for right cerebellum, and was 40.17% (SD = 6.415%) for right occipital lobe. Additionally, no significant gender differences in TMS coil-scalp distance (*t*_(21)_ = −0.769, *p* = 0.450), slope (*t*_(21)_ = 1.068, *p* = 0.298), and MT (*t*_(21)_ = 1.158, *p* = 0.26) were found.

### Accuracy Data

Overall, participants made significantly more errors in TMS trials compared to non-TMS trials. For stimulation of the right cerebellum, the mean error rate for non-TMS trials was 13.5% (SD = 8.6%), and increased to 23.9% (SD = 15.4%) for TMS trials. For stimulation of the right occipital lobe, the mean error rate for non-TMS trials was 12.8% (SD = 7.9%), and increased to 16.2% (SD = 12.2%) for TMS trials.

**Figure 2:**
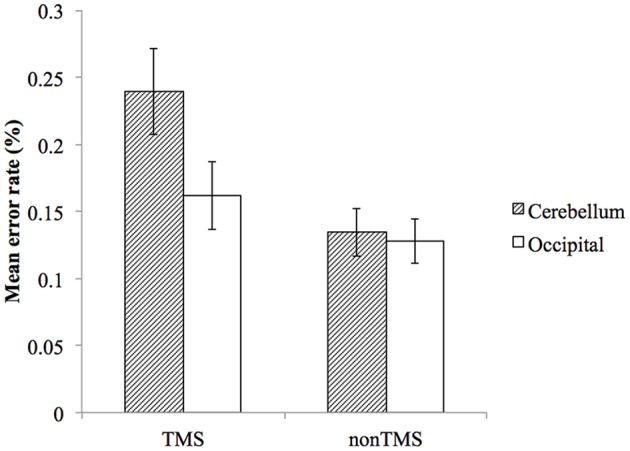
The effect of TMS on mean error rates for cerebellum and occipital lobe. Error bars represent SEM.

To examine whether the TMS effect on error rate is different between the two stimulation sites, we performed a repeated measure ANOVA with factors of *stimulation site* (cerebellum, occipital lobe) and *trial type* (TMS trial, non-TMS trial). This analysis yielded significant main effects of stimulation site (*F*_(1,22)_ = 5.719, *p* = 0.026), trial type (*F*_(1,22)_ = 10.845, *p* = 0.003), and a significant interaction effect (*F*_(1,22)_ = 6.333, *p* = 0.020), with cerebellar stimulation resulting in higher error rate than occipital lobe on TMS trials (Figure 2).

**Figure 3:**
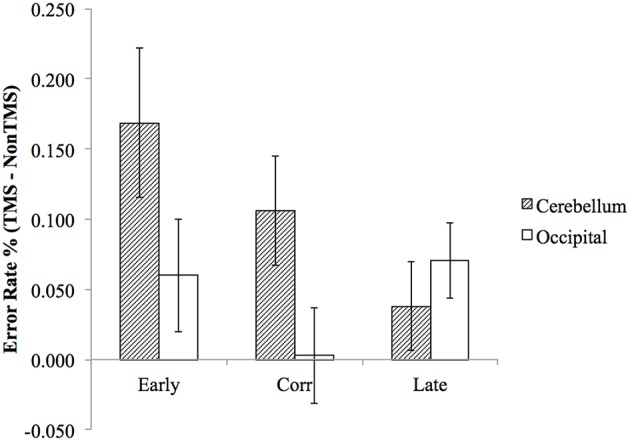
The difference in (TMS minus non-TMS) error rates between cerebellum stimulation and occipital lobe stimulation was significantly greater for “early probe” and “correct probe” condition, but not for the “late probe” condition. Error bars represent SEM.

To test our main *a priori* hypothesis that the early and correct probe would be more affected than the late probe by cerebellar TMS, we examined the error rate difference using a repeated measure ANOVA with factors of *probe position* (early probe, correct probe, late probe) and *stimulation site* (cerebellum, occipital lobe). To perform this analysis, we first calculated the mean error rate difference by subtracting the error rate of non-TMS trial from TMS trials, and then entered them into the repeated measure ANOVA described above. Consistent with our hypothesis, the planned comparison of the interaction between probe positions and stimulation site confirmed that the cerebellar TMS relative to occipital TMS resulted in significantly higher error rate for early probe and correct probe, compared to late probe condition (*F*_(1,22)_ = 4.78, *p* = 0.04). Direct paired *t*-tests were then conducted to assess the difference in error rate between cerebellar TMS vs. occipital TMS for early probe condition (*t*_(22)_ = 1.877, *p* = 0.074), correct probe condition (*t*_(22)_ = 2.466, *p* = 0.022), and late probe condition (*t*_(22)_ = 0.435, *p* = 0.795), as illustrated in Figure 3. The ANOVA also revealed a main effect for stimulation sites (*F*_(1,22)_ = 5.393, *p* = 0.03), with higher error rate in cerebellar stimulation condition, a non-significant main effect for probe position (*F*_(2,44)_ = 1.645, *p* = 0.205), and an interaction effect that approached significance (*F*_(2,44)_ = 2.670, *p* = 0.08).

### Reaction Time Data

For the RT data, participants were slower on TMS trials (Mean = 750.888 ms, SD = 40.219 ms) compared to non-TMS trials (Mean = 712.993 ms, SD = 30.647 ms). For stimulation of the right cerebellum, the mean RT for non-TMS trials was 706.322 ms (SD = 130.218 ms), and increased to 762.302 ms (SD = 191.992 ms) for TMS trials. For stimulation of the right occipital lobe, the mean RT for non-TMS trials was 719.664 ms (SD = 192.461 ms), and increased to 739.474 ms (SD = 218.334 ms) for TMS trials.

To examine whether the TMS effect on mean RT was different with respect to stimulation site, we performed a repeated measure ANOVA with factors of *stimulation site* (cerebellum, occipital lobe) and *trial type* (TMS trial, non-TMS trial). This analysis yielded a significant main effect of trial type (*F*_(1,22)_ = 5.805, *p* = 0.025) but not *stimulation site* (*F*_(1,22)_ = 0.029, *p* = 0.867), and there was no significant interaction (*F*_(1,22)_ = 2.569, *p* = 0.123). Thus, participants were significantly slower on TMS trials, but there was no difference in RT between TMS applied over cerebellum vs. occipital lobe.

We also examined whether the mean RT difference between TMS vs. non-TMS trials differed by *probe position* (early probe, correct probe, late probe) and *stimulation site* (cerebellum, occipital lobe). To perform this analysis, we first calculated the mean RT difference by subtracting the RT of non-TMS trials from TMS trials, and then entered them into a repeated measure ANOVA. This analysis yielded a significant main effect of probe position (*F*_(2,44)_ = 3.431, *p* = 0.042), but not stimulation site (*F*_(1,22)_ = 0.879, *p* = 0.359), and there was no significant interaction (*F*_(2,44)_ = 0.305, *p* = 0.687). Upon examination of the mean RT difference (TMS RT—nonTMS RT), the correct probe has the greatest RT difference (Mean = 59.338 ms, SE = 17.673 ms), followed by late (Mean = 37.122 ms, SE = 30.257 ms), and finally the early probe (Mean = −9.314 ms, SD = 20.223 ms).

## Discussion

We found that TMS administration to the right cerebellum, applied during covert rehearsal of a remembered sequence of letters, resulted in an interference with participants’ ability to identify whether a probe letter is in the correct position. Importantly, the pattern of results suggested that the response to the probe was based on out of date information regarding the next letter in the sequence. As a concrete example, in Figure 1, if TMS briefly causes the sequence prediction to be “frozen” at the letter B, then this letter b prediction will still be active when the probe letter is presented, and a probe letter of “b” will seem to be correct, whereas the actual correct probe of “r” will not, leading to errors on both the “early probe” and “correct probe” conditions. In contrast, “late probe” letters should still seem to be incorrect after TMS, and thus judgment of these letters should not be affected. The pattern of results depicted in Figure 3 supports this explanation and is consistent with our hypothesis that TMS pulses temporarily disrupt the function of the right cerebellum, resulting in prediction errors in the phonological loop. Our results therefore provide further evidence for cerebellar forward models in cognitive domains, in particular with respect to verbal working memory.

Our findings support the idea that cerebellar forward models contribute to verbal working memory by predicting upcoming verbal items in the phonological loop. In the motor control domain, the cerebellum is critical for predicting the outcome of an action before sensory feedback is available. These predictions can be compared with reafferent input. When they mismatch, an error signal is generated which allows rapid adjustments to the motor output as well as an update of the predictive model to refine future sensory predictions (Wolpert and Miall, 1996; Wolpert et al., 1998). Here, we presented evidence that the predictive capability of the cerebellum can be extended to verbal working memory. We proposed a cerebellar forward model that rapidly engages the phonological loop by computing an articulatory trajectory of the phonemes during the encoding phase. During the maintenance phase, the predicted output of the rehearsal process needs to be constantly compared to the content in the phonological store, which holds the correct sequence of phonemes kept in working memory. In previous studies, the encoding-related articulatory control process has been linked to right superior cerebellum *via* connection with Broca’s area and premotor cortex, while the maintenance-related phonological loop has been linked to right inferior cerebellum *via* connection with left inferior parietal lobule (Chen and Desmond, 2005). In the current study, TMS was administered to the right cerebellum during the guided rehearsal process. Since cerebellar sub-regions cannot be clearly delineated with TMS techniques, the significantly higher error rate could be a result of a compromised predictive process in the phonological store, the articulatory control system, or both. Interestingly, a recent functional magnetic resonance imaging (fMRI) study found activity in the right posterolateral cerebellum correlated with the predictability of upcoming sentence content, and the same cerebellar cluster that is sensitive to linguistic predictability was recruited in a phonological task, but not in semantic or orthographic tasks (Lesage et al., 2017). These results are consistent with our current findings and are in line with the idea that cerebellum plays a predictive role in verbal working memory and in language comprehension through prediction of phonological information.

Although our results provide further evidence for a forward model account of the cerebellar role in verbal working memory, there is no consensus regarding the basic function that the cerebellum provides for cognition, and other accounts such as the timing hypothesis (Keele and Ivry, 1990; Tesche and Karhu, 2000; Ivry et al., 2002; Leggio et al., 2011), and the sequencing hypothesis (Tesche and Karhu, 2000; Leggio et al., 2011), have also been proposed. According to the timing hypothesis, the cerebellum is essential for the representation of temporal relationships. In our experiment, the probe occurs at the fifth position in a six-letter sequence on 80% of the trials. Therefore, it is possible that participants developed a temporal prediction of the occurrence of the probe stimulus, and that application of TMS disrupted the internal timing component, resulting in an increased error rate. However, under a timing hypothesis, we would expect all probe types to be equally affected by cerebellar TMS. Our data clearly showed that the error rate significantly increased in the early and correct probe conditions, but not in the late probe condition. Hence, the pattern of results may be better understood in the context of forward models. Another putative cerebellar function is sequence detection, which emphasizes the cerebellum’s ability to detect and simulate repetitive sequence. It has been suggested that sequence detection is closely related to the predictive function characterized by forward models: the cerebellum creates internal models based on the sequence of events it detects (Leggio and Molinari, 2015). In verbal working memory, the “sequence” simulated by the internal model can be the content in the phonological store or the intended articulatory trajectory for rehearsal, which are respectively compared with the actual output of sub-vocal articulation or the actual trajectory of rehearsal. Therefore, the sequencing hypothesis is compatible with forward model explanations, and complements our findings of the functional role of cerebellum in verbal working memory.

Our data revealed a significant increase of RT in TMS trials compared to non-TMS trials. However, the RT difference was not significantly different between the stimulation sites (cerebellum vs. occipital lobe). In addition, the TMS effect on RT was not modulated by probe types (early, correct, late probes) between the two stimulation sites. This pattern of RT results, together with the significant TMS effects on accuracy, indicate that TMS interferes with the content in verbal working memory, rather than the speed of processing. These results are seemingly in conflict with a previous study showing cerebellar TMS resulted in an increase in RT during verbal working memory performance, but had no effect on accuracy (Desmond et al., 2005). However, a closer look of the task design in the previous study revealed that the TMS was administered immediately after encoding when the demand for preparation of articulatory trajectory is highest. On the other hand, in the current task, the TMS was administered closer to the end of the guided rehearsal phase when phonological store demand is highest. Given the known frontal/superior cerebellum circuit for articulatory preparation, and parietal/inferior cerebellum circuit for phonological store (Desmond et al., 1997, 2003, 2005), the TMS RT effect may likely reflect a compromised frontal/superior cerebellar articulatory control system, and the TMS accuracy effect may likely reflect a compromised parietal/inferior cerebellar phonological storage system. Taken together, the fact that we observed a TMS effect on accuracy (i.e., the content in working memory was affected), but not RT (i.e., processing speed was not affected), provide additional support that the right cerebellum plays a role in non-motor aspects of verbal working memory, and that a cerebellar forward model could explain the contribution of the cerebellum to non-motor cognitive functions, such as phonological storage.

In conclusion, our results are consistent with the idea that the right cerebellum supports verbal working memory by predicting upcoming verbal items in the phonological loop. It is assumed that a predictive process similar to forward models in motor control can be extended to non-motor cognitive functions such as verbal working memory, and the present study is consistent with other recent neuromodulation investigations supporting forward models in predicting verbal content (Lesage et al., 2012; Miall et al., 2016; D’Mello et al., 2017). Given the converging evidence from neuroimaging and anatomical studies, we speculate that: (1) the right superior cerebellum receives an “efference copy” of the articulatory command from Broca’s area, from which it generates a predicted articulatory trajectory of the encoded phonemes; and (2) the right inferior cerebellum receives an “efference copy” of the refresh phonological store command from the temporal/inferior parietal lobe, from which it generates a phonological trajectory of phonemes for active rehearsal. These predictions would then feedback to their respective cortical areas for speedy and accurate processing of phonological information in verbal working memory.

## Data Availability

The datasets generated for this study are available on request to the corresponding author.

## Author Contributions

JD conceived the presented idea and supervised the project. JD and Y-SS designed the experiment, analyzed the data and wrote the manuscript. Y-SS and YL carried out the experiment. Y-SS took the lead in writing the manuscript with support from JD.

## Conflict of Interest Statement

The authors declare that the research was conducted in the absence of any commercial or financial relationships that could be construed as a potential conflict of interest.

## Footnotes

^1^http://InstaMorph.com
